# How do the activity patterns of people with chronic pain influence the empathic response of future health professionals: an experimental study*

**DOI:** 10.1007/s10459-023-10291-2

**Published:** 2023-10-04

**Authors:** Rosa Esteve, Elena R. Serrano-Ibáñez, Sheila Castillo-Real, Carmen Ramírez-Maestre, Alicia E. López-Martínez

**Affiliations:** 1 Facultad de Psicología y Logopedia, Universidad de Málaga, Andalucía Tech, c/ Dr. Ortiz Ramos, 29010 Málaga, Spain; 2 Instituto de Investigaciones Biomédicas de Málaga (IBIMA), Málaga, Spain

**Keywords:** Activity patterns; chronic pain; empathy; health professionals; undergraduatesvignettes

## Abstract

Empathy in healthcare professionals is associated with better treatment outcomes and higher satisfaction among patients with chronic pain. Activity patterns play an essential role in the adjustment of these patients and, as a pain behaviour, may have a communicative function and elicit distinct empathic responses. This study investigated whether the activity pattern profiles characteristic of these patients had differential effects on the empathic response (empathic distress and compassion/sympathy) of future healthcare professionals. Healthcare professionals should improve their knowledge about the role of different activity patterns in the well-being of people with chronic pain and receive specific training in empathic skills. We controlled for several variables that could affect the empathic response (sex, age, academic degree, previous experience of chronic pain, and dispositional empathy).

A total of 228 undergraduates performed an experimental task using vignettes depicting four activity pattern profiles displayed by people with chronic pain and completed questionnaires measuring dispositional and situational empathy. We conducted a MANCOVA analysis.

Undergraduates showed more compassion/sympathy toward the medium cycler profile than toward the doer profile. Participants’ age was associated with empathic distress. Sex, academic degree, and previous experiences with chronic pain were not associated with their empathic response to the vignettes. Dispositional perspective-taking and empathic concern were significantly associated with compassion/sympathy responses, and personal distress was significantly associated with empathic distress.

Activity pattern profiles may have a communicative function and elicit different empathic responses toward people with chronic pain. Individual differences in dispositional empathy play an important role on situational empathic responses.

**Supplementary Information:**

The online version contains supplementary material available at 10.1007/s10459-023-10291-2.

## Introduction 

### Background

Communication between healthcare providers and patients with chronic pain is essential to the patients’ well-being (Matthias et al., 2010). When physicians are perceived as empathic by patients with chronic pain, they experience pain relief and improved quality of life (Cánovas et al., 2018). Moreover, low empathy among health professionals is related to adopting culturally negative stereotypes toward people with chronic pain and their stigmatization (Cohen et al., 2011). In the setting of pain, empathy is defined as *“a sense of knowing the experience of another person, with cognitive, affective and behavioural components”* (Goubert et al., 2005, p. 285).

Nevertheless, communication is not always comfortable. Physicians and patients with chronic pain indicate that the believability of the patient’s symptoms can hinder effective communication and cause distress to both parties (Kenny, 2004). On the one hand, doctors’ concerns relate to the credibility of the patients’ accounts of their pain, which is sometimes inconsistent with diagnostic test results (Matthias et al., 2010). Conversely, patients with chronic pain strive to be understood and believed by their doctors and “*not to appear too strong or too weak, too healthy or too sick”* (Werner & Malterud, 2003, p. 1409). Thus, as highlighted in the Communal Model of Pain Catastrophising (Sullivan, 2012), pain behaviours have a communicative function, and people with chronic pain use pain behaviours to regulate the interpersonal setting. In this line, a study of a sample of women diagnosed with fibromyalgia found that the avoidance of activity was related to the fear of negative evaluation by others (Écija et al., 2022). Furthermore, a recurrent concern among people with chronic pain is that family and friends do not understand their condition and blame them for “being lazy” and “sleeping all the time” (Turk et al., 2008).

Theoretical models of empathy and pain have established that empathic responses toward people with chronic pain are constructed through top-down influences (the observer’s learning experiences, goals, and pain-related beliefs) and bottom-up influences (contextual pain cues and the observation of pain behaviours and expressions) (Goubert et al., 2013). Among pain behaviours, the role of activity patterns in people with chronic pain has received increasing attention in recent years (Cane et al., 2018). We define activity patterns as consistent ways of organizing one’s occupations (Bendixen et al., 2006). People with chronic pain usually modify their activity to decrease pain and maximize their functioning (Racine et al., 2018). Research has identified several activity pattern profiles in people with chronic pain (Esteve et al., 2017; McCracken & Samuels, 2007): (a) avoiders: characterized by abandoning life activities because of chronic pain and interrupting actions when they think it will hurt; (b) doers: characterized by doing too much, doing more activities when they feel less pain, and not stopping motivating or valued tasks until they finish them; (c) extreme cyclers: characterized by doing too much and experiencing the rebound effects of heightened activity levels that make them avoid activity; and (d) medium cyclers: characterized by dividing daily activities into smaller tasks, taking frequent short rests, and slowing down so that they can do more things, reduce pain, and save energy to do other things that matter to them. These patterns are differentially related to adjustment (measured by positive and negative affect, daily functioning, and disability). The most adaptive profiles were doers, followed by medium cyclers, then extreme cyclers, and finally, avoiders (Esteve et al., 2017).

To the best of our knowledge, there is no research on the empathic response elicited by different activity patterns displayed by people with chronic pain. However, one study showed that family caregivers had a poorer estimation of their relative’s pain intensity (empathic accuracy) when patients presented higher physical functioning (i.e., the ability to perform daily activities) (Suso-Ribera et al., 2019). Another study found that when relatives and friends of people with chronic pain read vignettes describing characters experiencing pain, they estimated that pain was high when the characters reported severe pain and stopped all tasks when in pain. Conversely, they estimated pain and fairness as lowest when characters stopped disliked tasks but continued with liked tasks (Kappesser & Williams, 2008). One other study found that a large percentage of general practitioners and physical therapists believed that avoidance of activity was the most suitable pattern when patients were in pain and that they advised them according to these beliefs (Linton et al., 2002). Similarly, there is no research on the effects of activity patterns displayed by people with chronic pain on the empathic responses of Health Science students; however, it is essential to study their empathic responses because the development of empathy during their training period plays a critical role in their subsequent professional careers (Neumann et al., 2011; Nunes et al., 2011).

### Study aims

This study investigated whether the distinct activity pattern profiles displayed by people with chronic pain have differential effects on the empathic response of future healthcare professionals (i.e. undergraduate students following degrees in Medicine, Nursing, Occupational Therapy, Physiotherapy, Podiatry, or Psychology). We postulated that activity patterns would affect the empathic response; specifically, that the undergraduates would show more empathy toward people displaying an avoider profile than toward those displaying a doer profile. Our consideration of the extreme cyclers’ and medium cycler’s profiles was exploratory. We controlled for several top-down influences (Goubert et al., 2005) that could be associated with the empathic response (Archer et al., 1981; Eklund et al., 2009; Fields et al., 2011; Hojat et al., 2002), including sociodemographic variables such as sex, age, the academic degree, previous personal experience of chronic pain, and dispositional empathy.

## Methods

### Participants

The inclusion criteria were as follows: (a) being a 3rd- to 5th-year Health Science undergraduate at Málaga University (i.e., following degrees in Medicine, Nursing, Occupational Therapy, Physiotherapy, Podiatry, or Psychology); (b) understanding the Spanish language. We chose this range of courses to ensure that the students had carried out clinical internships that would have allowed them to contact patients with physical diseases that could be associated with chronic pain.

In total, 228 students participated in this study. Taking into account the number of experimental groups, this sample size is sufficient to detect significant medium effects (0.25) with high statistical power (0.80) at a significance level of 0.05 (Cohen, 1988).

### Variables and instruments

#### Sociodemographic variables

We asked the participants about their age, sex, the degree they were studying, and the course.

#### Contact with people with chronic pain

The assessment protocol included two yes-no questions asking the participants if they had carried out clinical curricular or extra-curricular practices or volunteering activities that had allowed them to be in contact with people with chronic pain. Furthermore, in another section, four dichotomous questions (yes-no) asked them about their close contact with relatives or patients with chronic or if they had chronic pain. If the participants answered “yes” to at least one question in each section we considered that they had personal experience of chronic pain or had been exposed to this through their professional practice.

#### Situational empathy

We assessed situational empathy using questions developed by Karos et al. (2018) based on Batson’s (1991) theoretical model. The participants were asked how they felt about the person described in the vignette by rating four self-oriented adjectives assessing empathic distress (worried, upset, anxious, sad) and three other-oriented adjectives assessing compassion/sympathy (understanding, compassionate, sympathizing). We used an 11-point Likert scale (ranging from 0 = ‘not at all’ to 10 = ‘very much’). Scores could range from 0 to 30 for compassion/sympathy and 0 to 40 for empathic distress, with higher scores indicating higher levels of compassion/sympathy and empathic distress, respectively. The internal consistency indexes were appropriate for both scales (compassion/sympathy, α = 0.80; empathic distress, α = 0.68).

#### Dispositional empathy

We applied the Spanish version of the Interpersonal Reactivity Index (IRI; Pérez-Albéniz et al., 2003), which is a 28-item instrument answered on a 5-point Likert scale ranging from “Does not describe me well” to “Describes me very well.“ The measure has four subscales: (a) perspective-taking, which evaluates a person’s ability to spontaneously put themselves in another’s place and adopt their perspective or psychological point of view; (b) fantasy, which analyses a person’s tendency to imaginatively identify with the feelings and actions of fictitious characters in movies, books, or plays; (c) empathic concern, which examines a person’s tendency to experience worry or feelings of compassion toward unfortunate others; and (d) personal distress, which assess a person’s tendency to experience feelings of anxiety or discomfort when witnessing others’ negative experiences. Higher scores indicate higher levels of empathy. The Spanish adaptation showed adequate reliability and validity, which were similar to that of the original instrument (Pérez-Albéniz et al., 2003). In the present study, the internal consistency indexes of the subscales were similar to those of the adaptation study (perspective-taking, α = 0.66; fantasy subscale, α = 0.80; empathic concern, α = 0.66; and personal distress, α = 0.75).

#### Experimental task

We used vignettes to present the four different activity patterns. Vignettes are short texts describing a specific person, an object, or a situation in detail. We followed the recommendations of Evans et al. (2015) to ensure the vignettes’ reliability and validity. The texts were written in a clear and precise style and adopted a neutral stance concerning cultural and socioeconomic factors; furthermore, we portrayed people as “real” individuals rather than as a list of symptoms.

We constructed four vignettes which were identical except for the information regarding the individual’s activity pattern, which was the independent variable. The four vignettes comprise a brief description about how a person with chronic pain performed their daily activities (Supplementary material). We included the activity pattern profiles described by Esteve et al. (2017): avoiders, doers, extreme cyclers, and medium cyclers. The instruction to participants was as follows: “Please read the following text carefully and answer the questions below”.

### Procedure

We obtained ethical approval from the University of Málaga Research Ethics Committee before data collection (CEUMA: 4-2022-H). The procedures followed the Declaration of Helsinki (1964) and its later amendments (World Medical Association Ethic Unit, 2007). We asked the teachers of the Health Science degrees at Málaga University for their collaboration and made an appointment for data collection, which took place in the regular classrooms. Firstly, we informed the participants of the study aims and procedures. We presented the study to the participants as an inquiry into how future healthcare professionals perceive patients with chronic pain. They were assured of the confidentiality and anonymity of the information collected and asked for their voluntary participation. Students and teachers did not receive any economic or academic compensation for participating in the study. Secondly, the participants gave their written informed consent. Thirdly, each participant received one of the four activity pattern vignettes, which were assigned randomly. After reading the vignette, the students completed the situational empathy scale (Karos et al., 2018) regarding the person with chronic pain described in the vignette. Finally, the participants completed the Interpersonal Reactivity Index (IRI; Pérez-Albéniz et al., 2003), the sociodemographic questionnaire, and the questions on their experience of patients with chronic pain, whether personal, through volunteering, or through clinical practice. Data were collected between 7 and 15 March, 2022. The average duration of the task was 20 to 25 min.

### Design

We used an experimental inter-subject design in which the independent variable comprised four levels corresponding to each vignette that described one of the following activity patterns: avoider, doer, extreme cyclers, and medium cyclers. We assigned the vignettes randomly. The dependent variable comprised the responses to the situational empathy scale (compassion/sympathy and empathic distress). We included as covariates sex, age, academic degree, previous personal experience with chronic pain, and dispositional empathy.

### Statistical analyses

All analyses were conducted using SPSS 22.0 (SPSS; Chicago, IL, USA). Firstly, we examined the frequency of the item scores. Because the missing data rate was acceptable (2%), we replaced them with the average item score. Secondly, we computed the total scores of the study variables and tested their distributions for kurtosis, symmetry, normality, homoscedasticity, and multicollinearity. Given that several variables did not follow a normal distribution, we used the Box-Cox transformation (Atkinson et al., 2021) to normalize them. The Mahalanobis distance method showed that there were no multivariate outliers (Leys et al., 2018). Thirdly, we calculated descriptive statistics for the variables included in the study. Fourthly, we computed bivariate correlations between the subscales of both instruments using Pearson’s coefficient. We interpreted correlations following the guidelines proposed by Cohen (1988), wherein low correlations range from 0.10 to 0.29, moderate correlations range from 0.30 to 0.49, and high correlations range from 0.50 to 1. Fifthly, we calculated the internal consistency of the subscales of the instruments using Cronbach’s Alpha coefficient (Cronbach, 1951). Finally, we conducted MANCOVA to test for differences between the experimental groups in situational empathy, while controlling for the following covariates: sociodemographic variables (sex, age, and academic degree), knowledge of chronic pain through personal or professional experience as well as dispositional empathy (perspective taking, empathic concern, fantasy, and personal discomfort). The assumption of homoscedasticity of variance-covariance was not met under the Box test. Thus, we used Pillai’s Trace statistic, whose values range from 0 to 1: higher values indicate that the effects contributed more to the model. To determine the effect size, we used partial *η*^*2*^, where values of 0.01 indicate minor effects, values of 0.06 medium effects, and values of more than 0.14 indicate large effects. We calculated the statistical power using Cohen’s *d*, taking as reference values 0.2, 0.5, and 0.8 for small, medium, and large size effects, respectively. We used Bonferroni *post hoc* tests to correct for multiple comparisons.

## Results

### Description of the participants

Of the 228 undergraduates who comprised the total sample, 164 were women (71.93%) and 64 were men (28.07%). Their average age was 22.95 years (*DT* = 4.53). They were studying Medicine (26.3%), Psychology (21.5%), Nursing (17.1%), Podiatry (15.4%), Physiotherapy (10.5%), and Occupational Therapy (9.2%). In total, 138 individuals (60.50%) had had contact with people with chronic pain through practical or volunteering activities, and 136 (59.60%) participants through personal experience. In total, 58 participants responded to the avoider profile vignette, 52 to the doer profile vignette, 58 to the medium cycler profile vignette, and 68 to the extreme cycler profile vignette.

### Descriptive statistics and correlations between dispositional and situational empathy variables

We computed the means, standard deviations, and Pearson’s bivariate correlations between the normalized dispositional and situational empathy variables. Moderate positive correlations were found between fantasy and empathic concern, empathic concern and personal distress, and personal distress and empathic distress (see Table 1).

**Table 1:** Means, Standard Deviations and Pearson correlations between normalized dispositional and situational empathy variables (*N* = 228)

Variables	Range	*M (SD)*	1	2	3	4	5	6
Dispositional Empathy								
1.Fantasy	3.32–5.92	4.88 (0.59)	1					
2. Perspective-taking	3.74–5.48	4.81 (0.36)	0.10	1				
3. Empathic Concern	3.61–6.24	5.55 (0.40)	0.41**	0.20**	1			
4. Personal Distress	2.45–5.20	3.73 (0.55)	0.27**	− 0.11	0.35**	1		
Situational Empathy								
5. Empathic distress	1.41–6.32	4.44 (0.88)	0.15*	− 0.06	0.25**	0.35**	1	
6. Compassion/sympathy	1.41–5.48	4.83 (0.55)	0.06	0.23**	0.19**	− 0.00	0.06	1

*Note: M =* mean; *SD =* standard deviation; *p < .05; ** *p* < .01

### MANCOVA

The overall MANCOVA results showed that the main effects of the different activity patterns displayed in the vignettes did not significantly affect situational empathy [Pillai’s trace = 0.048, *F* (6, 430) = 1.770, *p* = .104, *η*^2^ = 0.024, *d* = 0.668]. However, when considering the situational empathy variables separately, we found that although the type of activity pattern did not significantly affect empathic distress (Table 2), it did have a small effect on compassion/sympathy. Specifically, *post hoc* analyses showed that more compassion/sympathy was elicited by medium-cyclers than by doers. Therefore, we confirmed the hypothesis that the type of activity pattern would affect the participants’ empathic response toward people with chronic pain; however, the results led us to reject the hypothesis that the undergraduates would show more empathy toward people displaying an avoider profile than toward those displaying a doer profile. Post hoc analyses only found statistically significant differences in compassion/empathy between doers and medium cyclers. However, as Table 2 shows, it is interesting to note that doers elicited the lowest levels of compassion/sympathy, lower than any other group. Regarding compassion/empathy, the four activity pattern profiles can be ordered (from highest to lowest) as medium cyclers, extreme cyclers, avoiders, and doers.

**Table 2:** Means, standard deviations, and ANOVAs for comparisons between experimental conditions on situational empathy (*N =* 228)

Variables	Avoiders *n* = 58 *M* (*SD*)	Doers *n* = 52 *M* (*SD*)	Medium Cyclers *n* = 58 *M* (*SD*)	Extreme Cyclers *n* = 60 *M* (*SD*)	*F* (3, 215)^1^	*P*	*η* ^*2*^	*d*
Empathic distress	4.51 (0.78)	4.43 (1.02)	4.44 (0.87)	4.40 (0.86)	0.722	0.540	0.010	0.203
Compassion/sympathy	4.84 (0.61)	4.66 (0.73)	4.95 (0.36)	4.86 (0.39)	2.872	0.037	0.039	0.681

^1^*Note*: Degrees of freedom.

Concerning the covariates, with the exception of fantasy, the dispositional empathy variables were significantly associated with situational empathy: perspective-taking [Pillai’s trace = 0.043, *F* (2, 214) = 4.854, *p* = .009, *η*^2^ = 0.043, *d* = 0.797], empathic concern [Pillai’s trace = 0.039, *F* (2, 214) = 4.303, *p* = .015, *η*^2^ = 0.039, *d* = 0.744], and personal distress [Pillai’s trace = 0.077, *F* (2, 214) = 8.905, *p* = .000, *η*^2^ = 0.077, *d* = 0.971]. Specifically, perspective-taking [*F* (1, 215) = 8.574, *p* = .004, *η*^2^ = 0.038, *d* = 0.830] and empathic concern [*F* (1, 215) = 6.340, *p* = .013, *η*^2^ = 0.029, *d* = 0.708] were significantly associated with compassion/sympathy responses, with high statistical power and a small effect size. Personal distress [*F* (1, 215) = 17.128, *p* = .000, *η*^2^ = 0.074, *d* = 0.985] was significantly associated with empathic distress, with high statistical power and a medium-high effect. Finally, the participants’ age [Pillai’s trace = 0.033, *F* (2, 214) = 3.602, *p* = .029, *η*2 = 0.033, *d* = 0.662] was significantly associated with situational empathy; specifically, empathic distress [*F* (1, 215) = 7.202, *p* = .008, *η*^2^ = 0.032, *d* = 0.762]. The remaining covariates included in the model (fantasy, sex, academic degree, and contact with people with chronic pain through personal experience, curricular practice, extra-curricular practice, or volunteering activities) were not significantly associated with situational empathy.

## Discussion

This study investigated whether the distinct activity pattern profiles displayed by people with chronic pain had differential effects on the empathic response of Health Science undergraduates. We postulated that undergraduates would show more empathy toward people displaying an avoider profile than toward those displaying a doer profile. However, the hypothesis was rejected because we found that a higher level of compassion/sympathy was elicited by medium-cyclers than by doers.

Contrary to expectations, undergraduates did not show more empathy toward the avoider profile. We formulated this hypothesis according to previous research on empathic accuracy (Kappesser & Williams, 2008; Suso-Ribera et al., 2019), given that there was no specific research on the empathic response elicited by different activity patterns. Their results showed that family caregivers judged pain intensity to be lower when people with chronic pain had better daily functioning (Suso-Ribera et al., 2019). Conversely, in a vignette study, family caregivers estimated pain to be high when the characters reported that they stopped all tasks when in pain (Kappesser & Williams, 2008). Linton et al. (2002) found that a large percentage of general practitioners and physical therapists believed that the most suitable response on the part of their patients was to avoid activity when they were in pain. One possible explanation for these results is that current Health Science students receive better training on the deleterious effects of inactivity on health and that they do not hold fear-avoidance beliefs. Alternatively, it may be the case that the results of studies on the judgment of pain intensity by relatives and physicians are not generalizable to empathic responses.

Undergraduates showed more compassion/sympathy toward medium cyclers, who are characterized by pacing; they regulate their activity levels by dividing daily activities into smaller tasks, taking frequent short rests, and slowing down (Nielson et al., 2014). According to the results of previous studies, medium cyclers, after doers, have the most adaptive profile in terms of positive and negative affect, daily functioning, and disability (Esteve et al., 2017). We can speculate that future healthcare professionals felt more compassion/sympathy toward medium cyclers because of their academic and practical training. This possibility is supported by the finding that 90.2% of therapists working with patients with chronic pain teach them about pacing (Antcliff et al., 2019). Furthermore, implementing pacing behaviour is one of the main goals of the more traditional psychological interventions to promote adjustment to chronic pain (Scott-Dempster et al., 2017). Undergraduates seemed to value this activity pattern by which the character in the vignette functions despite pain and reorganises her activity.

The results obtained by Kappesser and Williams (2008) may explain the lower level of compassion/sympathy elicited by doers than by medium cyclers. Their vignette study showed that relatives and friends of people with chronic pain gave the lowest estimates of pain and fairness when the characters stopped disliked tasks but continued with liked tasks (Kappesser & Williams, 2008). They interpreted their results based on Social Contract Theory (Cosmides & Tooby, 2008). This theory states that individuals must pay a cost or meet a requirement to receive benefits and that the infraction of this rule implies enjoying the benefit without paying the corresponding cost or meeting the requirement (‘cheating’) (Cosmides & Tooby, 2008). In the present study, the doers’ vignette explicitly indicated that when the character started an activity that was motivating or essential to her, she continued until it was finished, regardless of whether she thought her pain would increase. In the light of the Social Contract Theory (Cosmides & Tooby, 2008), undergraduates in this study could have interpreted the doers’ profile as ‘cheating’ because the vignette character is not fulfilling her part of the social contract in the sense that she does not stop the activities she likes despite being in pain. However, we cannot test this aspect of the Social Contract Theory (Cosmides & Tooby, 2008) because, in this vignette study, we did not include a character that simultaneously stops disliked tasks and continues with liked tasks.

Finally, it is striking that our results showed that the different activity patterns did not affect the self-oriented responses (empathic distress) driven by the egoistic motivation to reduce personal distress. Conversely, they only affected other-oriented responses (compassion/sympathy), which may move people to an altruistic motivation to help others (Batson et al., 1991).

We controlled for several top-down influences (Goubert et al., 2005) that could affect the empathic response. We found that participants’ age was significantly associated with situational empathy: specifically, empathic distress. This result is in line with that of a previous study (Fields et al., 2011) of a sample of healthcare undergraduates, which found that empathy scores were higher among older students than among their younger classmates. In contrast with the results of previous studies (Eklund et al., 2009; Fields et al., 2011; Hojat et al., 2002), we found no associations between the situational empathy response and sex, academic degree, or previous personal experience with chronic pain.

The dispositional empathy variables, with the exception of fantasy, were significantly associated with the situational empathy responses. Specifically, perspective-taking and empathic concern were significantly associated with the compassion/sympathy responses, and personal distress was significantly associated with empathic distress. These results are in line with, and extend, the results of previous research on empathy in general (Archer et al., 1981; Davis, 1983) and pain-related empathy (Gourbert et al., 2008). They show that, besides situational factors, individual differences in empathy exert a significant influence on other- and self-oriented empathic responses.

The results of this study should be interpreted in the light of several methodological issues. Firstly, vignettes are helpful and well-established research tools in the social sciences; however, their ecological validity may be limited (Sampson & Johannessen, 2020). Even when participants respond honestly to vignettes, they may behave differently in real situations. Future research could improve the validity of the vignettes by presenting the fictional cases with audio-visual support. This approach would allow the observation of facial expressions, which are the most potent bottom-up influence on the empathic response to other people’s pain (Goubert et al., 2005; Xiong et al., 2019).

Secondly, there were more women than men in this study sample, which could explain the finding that there were no sex differences in the empathic response. However, this sample is an accurate representation of the sex ratio of students following Health Science degrees, at least in Spain, in which there are far more women than men. Furthermore, there were differences in the number of participants from the different Health Science degrees. Future research using more representative samples is needed. It would also be of interest to compare students’ empathic responses to different activity patterns to those of professionals with different years of experience.

Thirdly, it should be taken into account that although the effect of the different activity patterns on the undergraduates’ empathic responses was significant, its effect size was small. Fourthly, we treated the variable relating to contact with people with chronic pain (through personal or professional experience) as dichotomous (yes/no). This approach may have resulted in a loss of information and hindered the identification of other possible effects that previous experiences with people with chronic pain could have had. Future research could be conducted to investigate whether differences exist between the type of contact (through training or personal experience), duration, and chronic pain conditions. Finally, we used a general measure of dispositional empathy. Future studies could replicate this study by applying instruments that measure empathy among physicians, such as the Jefferson Scale adapted to Health Science students (Fields et al., 2011).

As far as we know, this study is the first to investigate whether the different activity pattern profiles characteristic of people with chronic pain influence the empathic responses of Health Science undergraduates. Despite its limitations, it opens a way to the study of the communicative function of activity patterns (Sullivan, 2012). Its results underscore the relevance of improving Health Science undergraduates’ knowledge of the role of different activity patterns in the well-being of people with chronic pain. Furthermore, this study highlights the need to provide specific training in empathic skills (Simko et al., 2021) to healthcare undergraduates and professionals caring for people living with chronic pain.

The results of this study have broader implications beyond the training of healthcare providers. The results are in line with those of previous research which have demonstrated the relevance of social interactions within the health-disease dichotomy (Cacioppo & Cacioppo, 2014; Holt-Lunstad et al., 2010; Wang et al., 2018). On the one hand, research has demonstrated how conflicts and interpersonal violence are associated with physical health problems such as chronic pain (López-Martínez et al., 2018). On the other hand, it has been suggested that social connection could protect and promote health (Feeney & Collins, 2015). Diary studies have shown that when people feel listened to and understood by others, they report more significant positive affect, fewer physical symptoms, greater satisfaction with life (Lun et al., 2008), and better pain adjustment (Sturgeon & Zautra, 2010). Furthermore, regardless of the level of perceived pain, people with chronic pain who have empathic relationships with others show higher resilience (Sturgeon & Zautra, 2010). As mentioned, activity patterns have a communicative function, which involves how others understand and assist the person with pain (Sullivan, 2012). Future research with other healthcare professionals and students, as well as family caregivers, should investigate whether people with chronic pain exhibiting a doer profile elicit less empathic responses and if these responses are associated, in the long run, with worse health outcomes. This line of research would open new venues of intervention in the relational sphere of chronic pain involving patients, healthcare providers, and caregivers. All of the above highlights the relevance of continuing to investigate how the interactions of activity patterns and interpersonal relationships are involved in the development and maintenance of health and illness, specifically in the population with chronic pain.

### Electronic supplementary material

Below is the link to the electronic supplementary material.

Supplementary Material 1
