# Supplementary material for: How do the activity patterns of people with chronic pain influence the empathic response of future health professionals: an experimental study*
Source: Adv Health Sci Educ Theory Pract. 2023 Oct 4;29(3):879–92. doi: 10.1007/s10459-023-10291-2 (PMC11208194; doi:10.1007/s10459-023-10291-2)
Supplement: Supplementary file 1 — Supplementary Material 1 [file 10459_2023_10291_MOESM1_ESM.docx]

**Appendix A. Supplementary material**

## Avoider’s pattern vignette

Julia has been suffering pain for several years. The pain is persistent and, as time passes, becomes more severe. She has consulted various doctors and does whatever she can to improve, but the pain remains. Julia says that for a long time she has not been able to carry on with her usual level of activity and that she has given up activities such as washing up, reading, shopping, or going out for dinner. She usually spends more time resting than doing activities. She says that if she is doing something such as cleaning thoroughly or going for a walk and thinks that her pain could increase, she stops the activity immediately.

## Doer's pattern vignette

Julia has been suffering pain for several years. The pain is persistent and, as time passes, becomes more severe. She has consulted various doctors and does whatever she can to improve, but the pain remains. Julia says that sometimes the pace of her activity is frenetic and, on the same day, she does the washing up, reads, goes shopping, and goes out for dinner. She usually makes the most of her good pain days by doing more things. Julia says that if she starts an activity that is motivating or essential to her, such as going for a walk or cleaning thoroughly, she keeps going until it is finished, regardless of whether she thinks her pain will increase.

## Medium cycler's pattern vignette

Julia has been suffering pain for several years. The pain is persistent and, as time passes, becomes more severe. She has consulted various doctors and does whatever she can to improve, but the pain remains. Julia says that the tasks she used to do on the same day, such as washing up, reading, shopping, or going out for dinner, she now distributes them over several days, so that she experiences less pain. Julia usually does things more slowly so that she can do a lot more things. She says that she takes several breaks to save energy to do other things that matter to her, such as cleaning thoroughly or going for a walk.

## Extreme cycler's pattern vignette

Julia has been suffering pain for several years. The pain is persistent and, as time passes, becomes more severe. She has consulted various doctors and does whatever she can to improve, but the pain remains. Julia says that sometimes the pace of her activity is frenetic and, on the same day, she does the washing up, reads, goes shopping, and goes out for dinner. She is usually exhausted the day after and spends more time resting than doing activities until she feels recovered. She says that, as time passes, she is giving up more activities that she used to do, such as cleaning thoroughly or going for a walk.
